# Leveraging biotin-based proximity labeling to identify cellular factors governing early alphaherpesvirus infection

**DOI:** 10.1128/mbio.01445-24

**Published:** 2024-07-02

**Authors:** Jenai Quan, Qing Fan, Lacy M. Simons, Samuel N. Smukowski, Caitlin Pegg, Richard Longnecker, Jeffrey N. Savas, Judd F. Hultquist, Gregory A. Smith

**Affiliations:** 1 Department of Microbiology-Immunology, Northwestern University Feinberg School of Medicine, Chicago, Illinois, USA; 2 Division of Infectious Diseases, Department of Medicine, Northwestern University Feinberg School of Medicine, Chicago, Illinois, USA; 3 Department of Neurology, Northwestern University Feinberg School of Medicine, Chicago, Illinois, USA; Duke University School of Medicine, Durham, North Carolina, USA

**Keywords:** HSV, PRV, tegument, pUL36, pUL37, proximity labeling, BioID2, CRISPR, zyxin, cytoskeleton

## Abstract

Neurotropic alphaherpesviruses, including herpes simplex virus type 1 and pseudorabies virus, establish a lifelong presence within the peripheral nervous system of their mammalian hosts. Upon entering cells, two conserved tegument proteins, pUL36 and pUL37, traffic DNA-containing capsids to nuclei. These proteins support long-distance retrograde axonal transport and invasion of the nervous system *in vivo*. To better understand how pUL36 and pUL37 function, recombinant viral particles carrying BioID2 fused to these proteins were produced to biotinylate cellular proteins in their proximity (<10 nm) during infection. Eighty-six high-confidence host proteins were identified by mass spectrometry and subsequently targeted by CRISPR-Cas9 gene editing to assess their contributions to early infection. Proteins were identified that both supported and antagonized infection in immortalized human epithelial cells. The latter included zyxin, a protein that localizes to focal adhesions and regulates actin cytoskeletal dynamics. Zyxin knockout cells were hyper-permissive to infection and could be rescued with even modest expression of GFP-zyxin. These results provide a resource for studies of the virus-cell interface and identify zyxin as a novel deterrent to alphaherpesvirus infection.

**IMPORTANCE:**

Neuroinvasive alphaherpesviruses are highly prevalent with many members found across mammals [e.g., herpes simplex virus type 1 (HSV-1) in humans and pseudorabies virus in pigs]. HSV-1 causes a range of clinical manifestations from cold sores to blindness and encephalitis. There are no vaccines or curative therapies available for HSV-1. A fundamental feature of these viruses is their establishment of lifelong infection of the nervous system in their respective hosts. This outcome is possible due to a potent neuroinvasive property that is coordinated by two proteins: pUL36 and pUL37. In this study, we explore the cellular protein network in proximity to pUL36 and pUL37 during infection and examine the impact of knocking down the expression of these proteins upon infection.

## INTRODUCTION

Neuroinvasive alphaherpesviruses inflict a significant burden on human health and the agricultural industry. Herpes simplex viruses (HSV-1 and HSV-2) infect 3.7 billion people globally (1). Although HSV infection is commonly asymptomatic, it can cause blindness (herpes simplex keratitis), life-threatening neurological disease (herpes simplex encephalitis), and severe infections in newborns and immunocompromised patients (2–4). Veterinary alphaherpesviruses that infect cattle, poultry, swine, and horses are a substantial economic burden. Of the latter, one of the most well studied is pseudorabies virus (PRV), which is a common model of alphaherpesvirus replication, pathogenesis, and neurovirulence (5). These viruses share a hallmark capacity to invade the nervous system by retrograde axonal transport and establish latent neuronal infections.

Alphaherpesvirus infection is a coordinated process mediated by viral effectors that hijack cellular proteins in a spatially and temporally regulated manner. During initial infection, prior to the onset of viral gene expression, structural components of the virion deliver the viral genome to nuclei. The virion houses the DNA genome within a proteinaceous capsid, which is surrounded by an additional tegument layer of proteins and a membrane envelope. Following fusion-based entry into the cell, the 125 nm diameter capsid and associated tegument proteins are deposited into the cytosol. Of the approximately 20 tegument proteins in the incoming particle, three are retained on HSV-1 and PRV capsids, which we collectively refer to as the post-entry capsid complex (PECC): pUS3, pUL36, and pUL37 (6–9). pUS3 promotes capsid delivery by disrupting the cortical F-actin that the virus encounters after fusion at the plasma membrane (10) but is otherwise dispensable for microtubule transport (11). pUL37 promotes the nuclear translocation of capsids by regulating the kinesin-1 microtubule motor and binds the cargo-microtubule linker protein, dystonin (12–16). Both pUS3 and pUL37 are tethered to the capsid by pUL36 (17, 18). pUL36 interacts with the microtubule motors dynein/dynactin and kinesin-1 (19, 20) and is essential for capsid microtubule-based transport (21). How these proteins perform these functions is not fully elucidated. Given that pUL36 and pUL37 are the only two viral proteins known to promote capsid transport on microtubules, we hypothesized that additional trafficking factors would be detectable by proximity labeling.

BioID2 is a promiscuous biotin ligase that catalyzes the addition of biotin to lysine residues in proteins within a roughly 10 nm radius. Biotinylated proteins can be captured by streptavidin pulldown and subjected to proteomic analysis. This method is advantageous in identifying weak, indirect, or transient interactions between proteins that may go undetected by other methods, such as affinity pulldown or co-immunoprecipitation (22, 23). This method has been useful in identifying previously unrecognized host factors for human immunodeficiency virus (HIV), severe acute respiratory syndrome coronavirus type 2, Kaposi’s sarcoma-associated herpesvirus, human cytomegalovirus (HCMV), and HSV-1, but it has not yet been used to characterize alphaherpesvirus tegument proteins during early infection (24–29).

In this study, we generated PRV strains encoding BioID2 fusions to either pUL36 or pUL37, which were used to identify 897 proximal protein partners during infection. Eighty-six high-confidence hits were tested for functional relevance in a CRISPR-Cas9 screen. This pipeline identified numerous cellular proteins that were in proximity to the PECC and that either antagonized or supported early infection, several of which were previously described. Several other putative host factors were novel, including zyxin, which was identified as an antagonist of early infection.

## RESULTS

### Proximity-labeling virus design and validation

**Fig 1:**
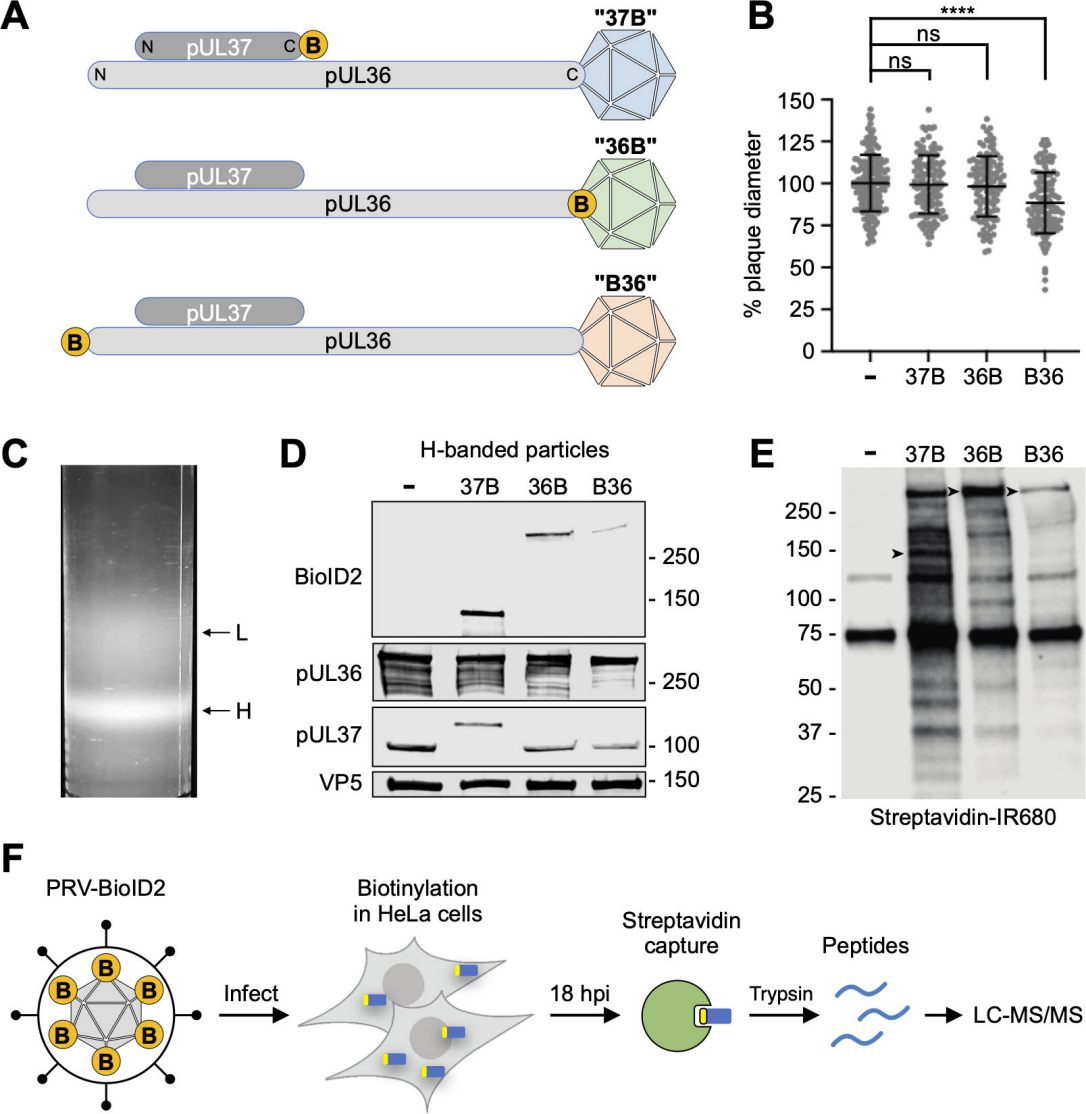
Production of PRV-expressing BioID2. (**A**) Schematic of three PRV-BioID2 designs that place BioID2 (yellow circles marked with “B”) at either the amino- or carboxyl-terminus of the indicated protein. (**B**) Plaque diameters relative to unmodified (−) PRV. ****P < 0.0001 (unpaired *t*-test). (**C**) Representative image of dextran gradient showing PRV light particles (L) and heavy particles (H), the latter of which are fully assembled virions. (**D**) H-banded extracellular virions probed for BioID2 and the indicated PRV structural proteins. (**E**) Streptavidin blot of HeLa cells infected for 18 hours. Black arrowheads indicate the predicted BioID2 fusion. (**F**) Schematic of proximity-dependent biotinylation and protein identification (blue, target cellular protein; yellow, biotin).

Three recombinants of PRV were produced that either incorporate the BioID2 probe (27 kDa) on the C-terminus of pUL37 (37B), the N-terminus of pUL36 (B36), or the C-terminus of pUL36 (36B) (Fig. 1A). This design strategy was based on previous observations that similar GFP (28 kDa) fusions were well-tolerated by PRV (7, 30, 31). The 37B and 36B viruses attained titers (5.5 × 10^8^ PFU/mL and 4.2 × 10^8^ PFU/mL, respectively) that were statistically equivalent to untagged PRV (mean titer: 5.8 × 10^8^ PFU/mL), while the B36 virus was slightly attenuated (1.2 × 10^8^ PFU/mL; *P* < 0.01). Similarly, the 36B and 37B viruses produced plaque diameters that were equivalent to the untagged virus, while average B36 plaque diameters were reduced by 12% (*P* < 0.0001) (Fig. 1B). To determine if the BioID2 fusion proteins were incorporated into virions, extracellular heavy particles (H-particles) were isolated by velocity sedimentation (Fig. 1C). The 27 kDa BioID2 fusion was detected in particles from the three PRV recombinants by immunoblot, but a mobility shift was not observed in the pUL36 fusions due to the latter’s mass (Fig. 1D). Fusion protein expression was also verified in infected cells (Fig. S1). To confirm that the BioID2 fusions were enzymatically active, HeLa cells were incubated with 20 µM biotin for 16–18 hours and infected at MOI:3. HeLa cells were used due to the available human proteomic reference and to allow future comparative studies with HSV. To understand how pUL36 and pUL37 function to traffic capsids to nuclei, we would ideally have examined biotinylation within 2 hours post-infection (hpi); however, BioID2 activity was insufficient to achieve this, and we instead prepared whole cell lysates at 18 hpi, which were blotted with streptavidin to confirm biotinylation had occurred (Fig. 1E). 37B infection resulted in the most biotinylated substrates, while B36 resulted in the fewest.

### Proteins identified by proximity labeling

To identify the proteins that were biotinylated by the BioID2 viruses, infections of HeLa cells were performed as above but the biotinylated proteins were captured by streptavidin pulldown (Fig. 1F). The biotinylated proteins were digested with trypsin, and the resulting peptides were analyzed with liquid chromatography-tandem mass spectrometry (LC-MS/MS)-based proteomic analysis (32).

**Fig 2:**
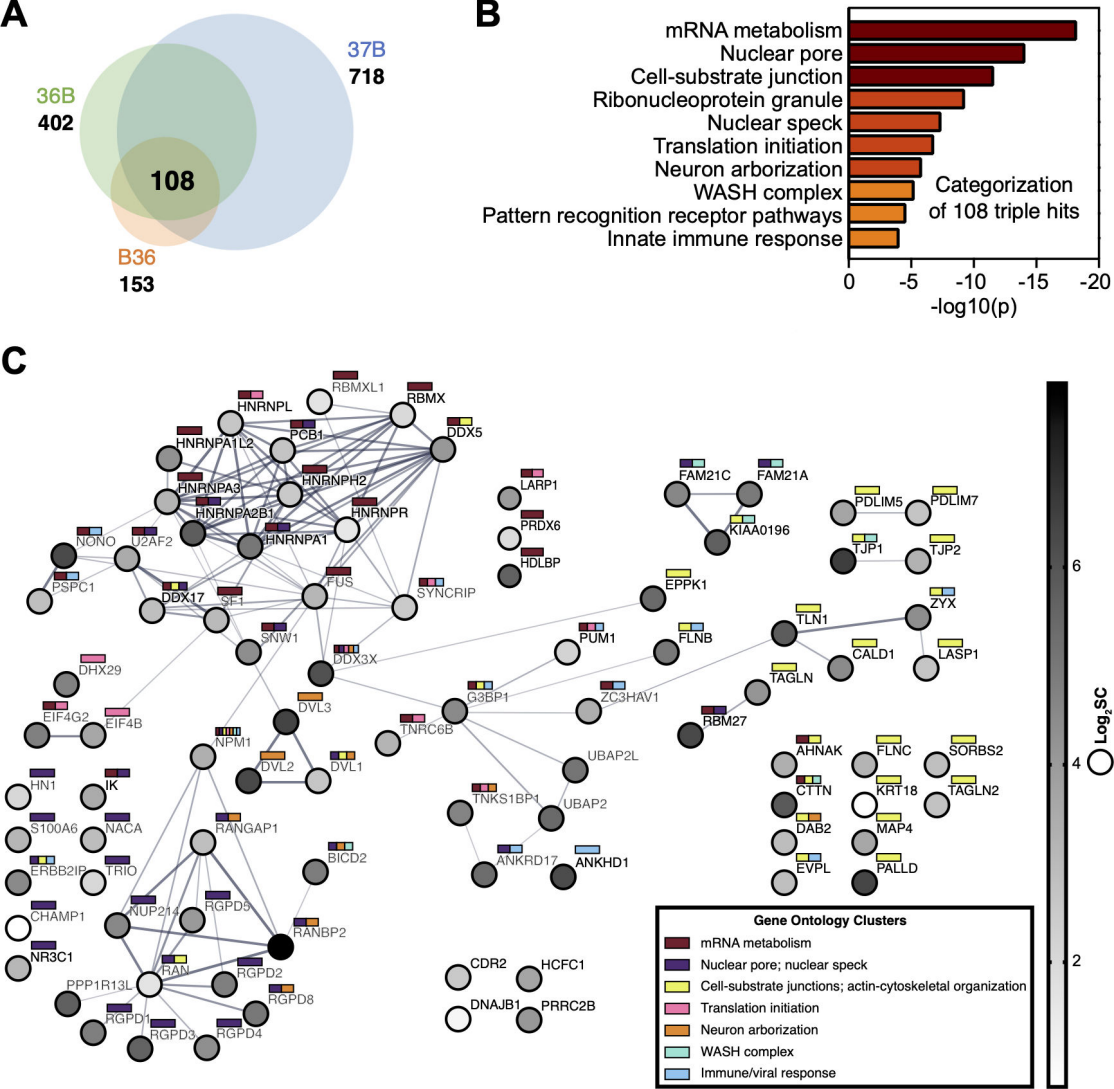
Proximity labeling of cellular proteins during PRV infection. (**A**) Venn diagram of the number of unique cellular proteins identified by the three BioID2 viruses that were absent from the untagged virus control. (**B**) Metascape-generated gene ontology of proteins identified by all three viruses. (**C**) STRING network and functional categorization of cellular proximity proteins. Nodes (cellular proteins) are shaded by the average log_2_-fold change spectral count across the three BioID2 viruses. Edges connecting nodes indicate a reported physical interaction, where thickness represents the interaction confidence (bold, high confidence). Node color tags determined by association with encompassing gene ontological classifications are provided at the top right of each node.

Collectively, peptides mapping to 2,953 unique protein identifiers were detected across all viruses and runs (File S1). After removing known contaminants, peak calling controls, and isoform duplicates, these were mapped to 1,311 human and 24 viral proteins. The unique viral proteins were detected by at least one of the three BioID2 viruses in the final run (File S2; Fig. S2). Of the human cellular proteins, 414 were detected in the untagged virus control (spectral count > 0) and were removed from the analysis, leaving a total of 897 cellular proteins (File S1). The number of unique cellular proteins identified by each virus corresponded with the intensity of the biotin labeling detected in the Western blot (Fig. 1E), including 718 identified with virus 37B, 402 with virus 36B, and 153 with virus B36. We focused on the 108 cellular proteins that were identified in proximity to all three viral probes with the rationale that these were most likely to be in proximity to the PECC (Fig. 2A).

Excluding ribosomal and histone proteins (*n* = 16), the remaining proteins were significantly enriched for several Metascape gene ontologies (GO) (Fig. 2B) (33). These included the nuclear pore ontology (GO:0005643) that also included members of the nucleic-acid transport ontology (GO:0050657), and the cell-substrate junction ontology (GO:0030055) that included members of the supramolecular fiber organizational ontology (GO:0097435). Ontologies involving antiviral host responses were also highly enriched and included pattern recognition receptor pathways (GO:0039531) and innate immune response (GO:0045087).

To better visualize the network of cellular proteins found in proximity to the PECC, we used the STRING database to identify putative protein interactions among the 88 top proximity hits (excluding the 16 ribosomal and histone proteins and four targets that were not mapped by STRING). Membership for each factor in the pertinent gene ontologies was overlaid with nodes shaded by average spectral count in the mass spectrometry results (Fig. 2C). This network classifies several high-confidence protein clusters that were enriched in the vicinity of the PECC and included components of the actin cytoskeleton, endosomal trafficking (e.g., the WASH complex), nuclear import, and antiviral responses.

### Design of a CRISPR screen for functional assessment of proximity candidates

**Fig 3:**
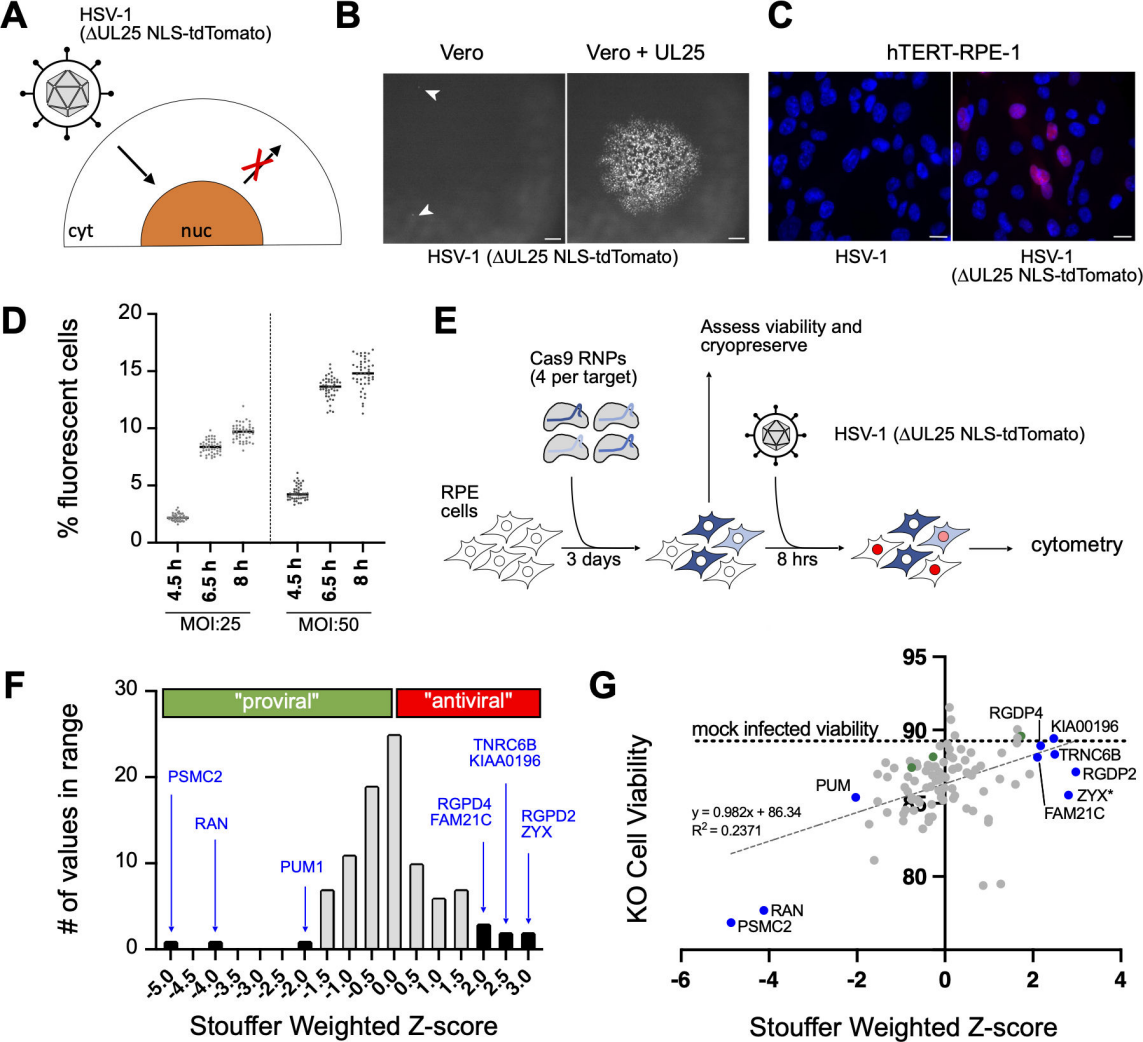
CRISPR screen to test the functionality of proximal proteins during early HSV-1 infection. (**A**) Schematic of the single-round immediate-early reporter HSV-1 that produces nuclear fluorescence upon nuclear genome delivery. (**B**) Wide-field fluorescence microscopy 3 days post-infection with the single-round reporter HSV-1 demonstrating single-cell infections (white carets) on standard Vero cells (left panel) and propagation in UL25-complementing Vero cells (right panel). Scale bars = 150 µm. (**C**) Wide-field fluorescence microscopy of nuclear fluorescence in RPE cells infected with MOI:5 at 8 hpi with the untagged control virus and the single-round reporter virus (blue, Hoechst nuclear staining). Scale bars = 20 µm. (**D**) Time course of nuclear fluorescence in infected RPE cells measured by flow cytometry (bars, medians). (**E**) Strategy for determining the functional significance of proximity cellular proteins. (**F**) Histogram of cell targets based on Stouffer’s weighted *Z*-scores. Cellular proteins meeting the threshold for significance of *Z* > |1.96| are indicated with blue text. (**G**) Average viability of polyclonal KO cells plotted against Stouffer’s weighted *Z*-scores. Best fit line is plotted in light gray dashed line. Mock-infected cell viability is plotted as dotted black line. Cellular proteins meeting the threshold for significance of *Z* > |1.96| are highlighted in blue. Samples that received non-targeting (scrambled) gRNA complexes are highlighted in green.

To assess if cellular proteins identified by the BioID2 viruses have a functional impact on viral genome delivery to nuclei, a single-round reporter virus expressing a fluorescent protein was used to infect hTERT-RPE1 (RPE) cells in a CRISPR-Cas9 knockout screen (Fig. 3A). A switch from PRV to HSV-1 for this portion of the study was decided upon for two reasons. First, because nuclear delivery is a fundamental initial step of infection for all alphaherpesviruses, hits from the PRV proximity screen that impacted HSV-1 nuclear delivery were expected to be of greatest interest. Second, for this functional screen, pairing a human virus with the human cells was desired. RPE cells, which infect well with HSV-1, were used due to the benefit that their normal ploidy would afford in the knock-out screen (34, 35). The reporter virus, HSV1-GS5451, encodes the tdTomato fluorescent protein fused to a nuclear localization signal driven by the immediate early CMV promoter (US5::CMVIE > NLS-tdTomato) and is deleted for the UL25 capsid gene to prevent secondary rounds of infection (36). HSV1-GS5451 was verified to propagate on UL25-complementing Vero cells but not on standard Vero cells, as demonstrated by the restricted single-cell infection in Vero cells (Fig. 3B) and the production of red-fluorescent nuclei by 6 hpi (Fig. 3C). The application of this virus to a reporter screen based on flow cytometry was validated with RPE cells in a 96-well infection format. We selected conditions that would achieve at least 10% infection in non-targeted cells to allow for a dynamic window of infection change from a 1-log-fold increase to a 1-log-fold decrease (Fig. 3D).

### Functional contribution of cellular targets toward infection

Of the 108 cellular protein candidates identified using BioID2 (Fig. 2A), 30 genes were excluded from the CRISPR screen due to genetic redundancy with known paralogs or because their depletion is known to affect cell viability (such as ribosomal proteins, histones, and nucleoporins). Eight additional targets that were consistently detected (SC > 0) in preliminary BioID2 optimization studies were included (File S1 ), bringing the total number of targets to 86. Three non-targeting guides (NT1–NT3) were included as negative controls. For the screen, RPE cells were electroporated with CRISPR-Cas9 ribonucleoprotein complexes (RNPs) in 96-well arrayed format (Fig. 3E). Each well contained four independent guide RNA designed to result in loss-of-function of the gene target. Cells were then replica-plated for infection with HSV1-GS5451 in triplicate, cell viability assessment, and cryopreservation. Infected cells were harvested at 8 hpi, and fluorescence was measured from each infection by flow cytometry. The log_2_-fold change in percent infected cells was calculated relative to the plate median and averaged across technical replicates before being converted to *Z*-scores. The screen was repeated, and Stouffer’s method was used to aggregate *Z*-scores across the two independently conducted screens (Fig. 3F; File S3).

None of the non-targeting controls were significant and clustered close to the median. Three targets produced a significant negative *Z*-score (<−1.96) indicating decreased infection upon CRISPR targeting. These three putative “proviral” targets were all previously reported to promote HSV-1 infection: Pumillo1 (PUM1), RanGTPase (RAN), and the proteasome 26S subunit (PSMC2) (37–39). On the other hand, six targets produced positive *Z*-scores (>1.96) that indicated increased infection upon CRISPR targeting: RANBP2-like and GRIP domain containing 2 and 4 (RGPD2 and RGPD4), trinucleotide repeat containing adaptor 6B (TNRC6B), FAM21C, KIA00196 (aka, strumpellin), and zyxin. To the best of our knowledge, none of these “antiviral” targets were previously studied in the context of HSV-1 infection. To ensure these changes were not driven by differences in cell viability, the infection rates to cell viability were compared. No correlation (*R*^2^ = 0.2371) between cell viability and infection as signified by aggregated *Z* scores was detected (Fig. 3G).

### Validating the contribution of WASH complex components to herpesvirus infection

Two components of the WASH complex, strumpellin and FAM21C, restricted HSV-1 infection in the CRISPR-Cas9 screen. To further validate their relevance, we attempted to isolate clonal populations of RPE cells lacking expression of either protein from the cryopreserved cells from the CRISPR screen; however, no full knockouts were identified for either target. As an alternative, RPE cells were transduced with CRISPR-Cas9 lentiviruses encoding gRNAs targeting either strumpellin (S-gRNA 1 through S-gRNA 4) or FAM21C (F-gRNA 1 through F-gRNA 4). The cells were transduced with individual lentiviruses or a pool and placed under puromycin selection. Unfortunately, depletion of these proteins was again incomplete in all attempts (Fig. S3A and B). Nevertheless, HSV-1 fluorescent reporter expression in these knock-down cells was reduced relative to cells transduced with a non-targeting gRNA lentivirus (Fig. S3C). The reduction was also observed with a replication-competent HSV-1 (Fig. S3D). While these results added additional support that components of the WASH complex antagonize HSV-1 infection, we focused our efforts on another target for the remainder of this study for which knockout cells were obtained.

### Validating the contribution of zyxin to early herpesvirus infection

**Fig 4:**
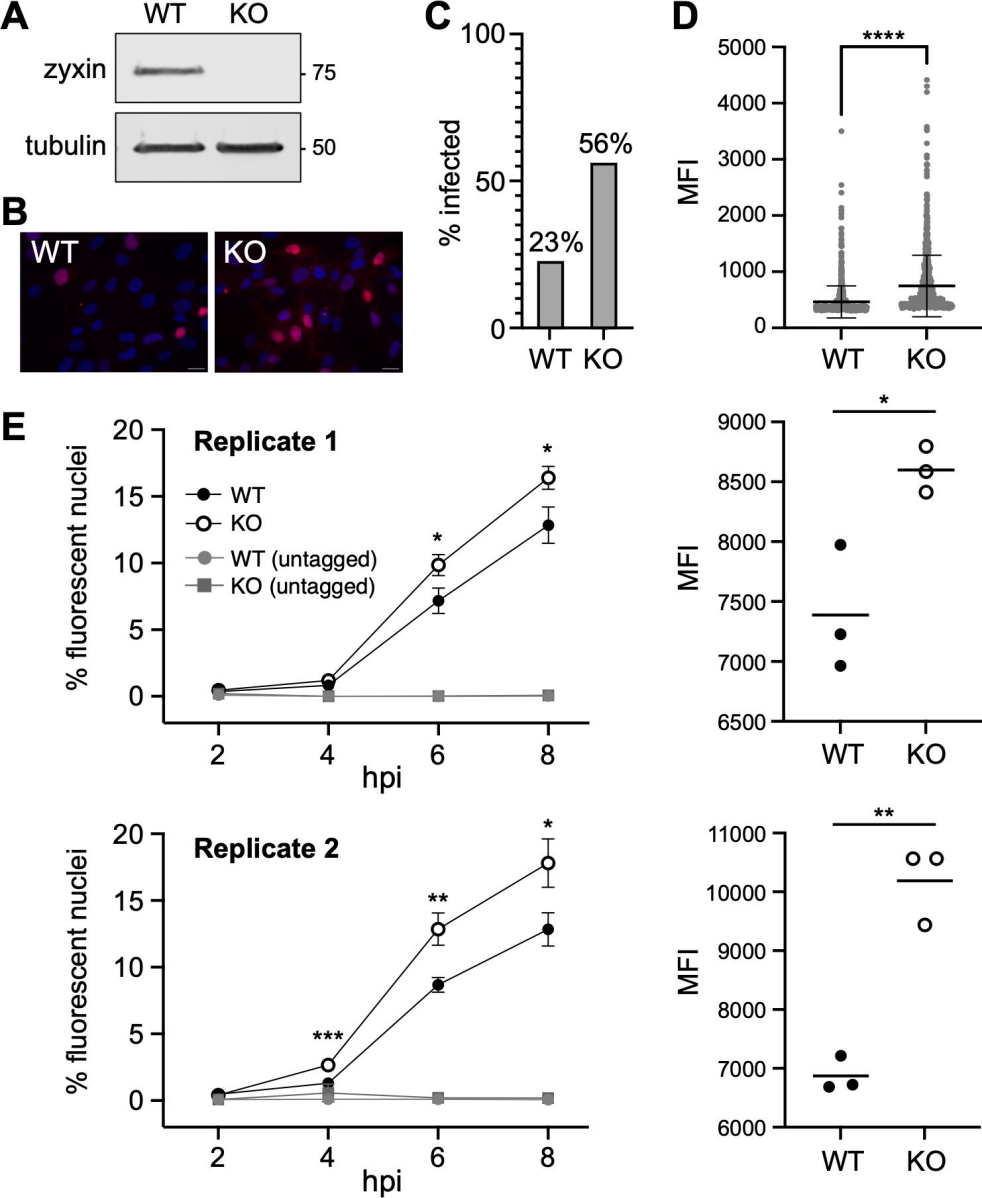
Depletion of zyxin increases permissivity to early infection. (**A**) Immunoblot of RPE lysates (WT and clonal zyxin KO). (**B**) Representative images of WT and zyxin-KO cells infected with the HSV-1 single-round reporter (MOI:30, 5 hpi). Scale bars = 20 µm. (**C**) Proportion of infected cells and (**D**) their nuclear mean fluorescence intensities based on images represented in panel B. ****P < 0.0001 (unpaired *t*-test). (**E**) Time course of fraction of cells emitting nuclear fluorescence (percent infected) analyzed by flow cytometry following infection with HSV-1 single-round reporter in WT and zyxin-KO cells. Two replica experiments, each done in triplicate, are shown along with their corresponding MFIs from 6 hpi. *P < 0.05, **P < 0.01, and ***P < 0.001 (unpaired *t*-test between WT and KO-tagged virus infection).

Zyxin was one of the strongest “antiviral” factors identified in our screen, and a clonal population of RPE cells lacking zyxin expression was recovered from the cryopreserved cells from the CRISPR screen (Fig. 4A). As a preliminary test, wild-type (WT) and zyxin-KO RPE cells were infected with HSV1-GS5451 and imaged by fluorescent microscopy at 5 hpi (Fig. 4B). Both the percentage of infected cells (Fig. 4C) and the median fluorescence intensity (MFI) from the infected cells (Fig. 4D) were increased in the zyxin-KO population. Next, we monitored the development of reporter gene expression between 2 and 8 hpi with HSV1-GS5451 or an untagged control HSV-1 by flow cytometry. This experiment was performed twice in biological triplicate (Fig. 4E). In the first replicate, 38% more zyxin-KO cells were emitting detectable fluorescence than WT cells at 6 hpi (*P* < 0.05), and the corresponding MFI of the infected KO cells was also increased (*P* < 0.05). Similar results were observed in the second replicate, with 48% more zyxin-KO cells emitting detectable fluorescence at 6 hpi (*P* < 0.05) and an associated MFI increase (*P* < 0.01). This boost in MFI trended from 4 to 8 hpi (Fig. S4). These data suggest that zyxin-KO cells are more susceptible to HSV-1 than wild-type RPE cells.

**Fig 5:**
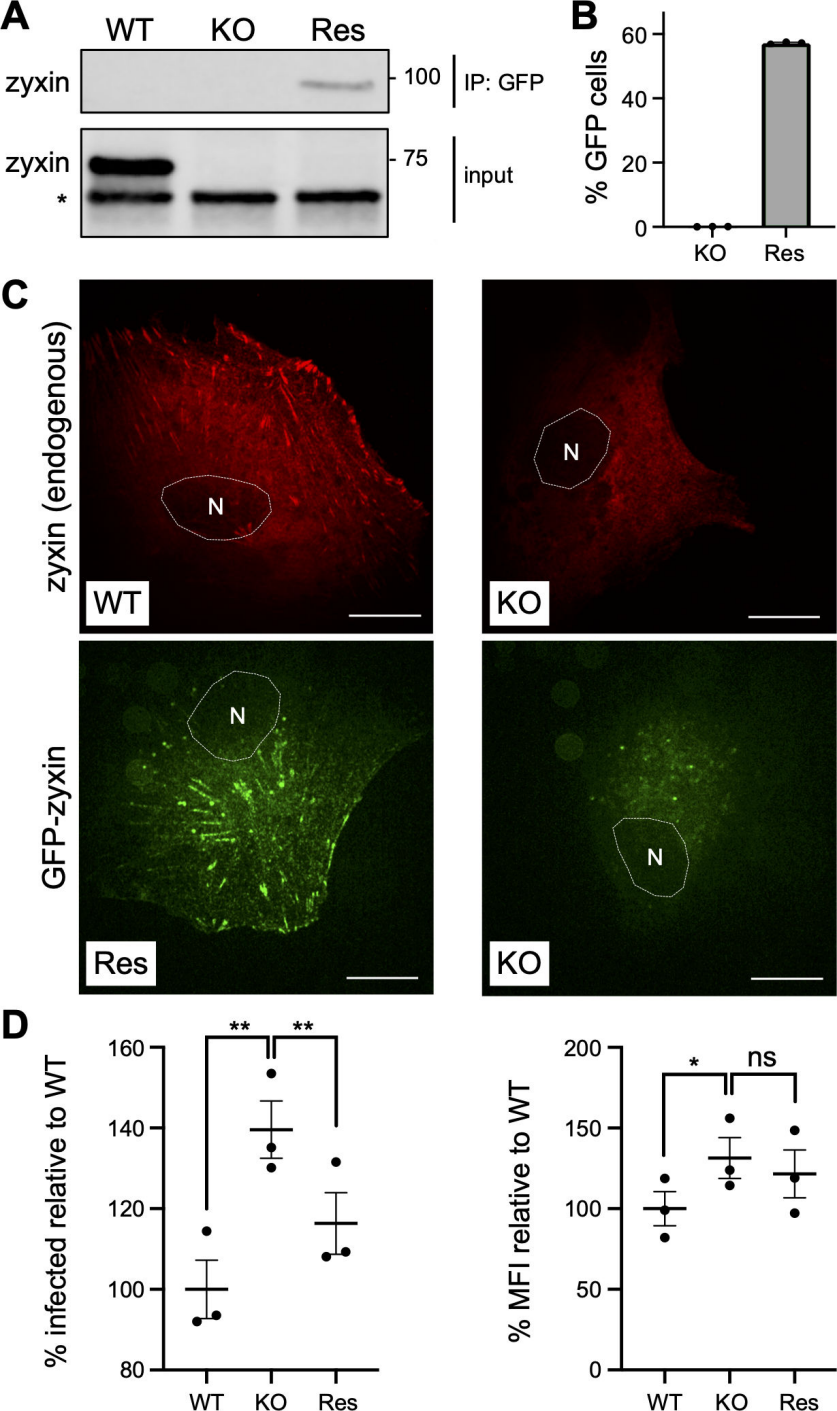
Rescue of zyxin knockout cells. (**A**) Immunoblot of zyxin from WT, zyxin-KO, and KO cells rescued with mouse GFP-zyxin (Res) (*non-specific band). (**B**) Flow cytometric determination of GFP-positive population in zyxin-KO and rescue cells. (**C**) Zyxin immunofluorescence (red) in WT and KO cells. GFP emissions from rescue and KO cells. Scale bar = 20 µm; gamma adjustments were applied to images equally. (**D**) Relative amounts of infected cells based on tdTomato expression from the HSV-1 single-round reporter at 6 hpi, with corresponding median fluorescence intensities at right. Each data point is the mean of medians from internal triplicates, with the overall mean indicated with standard error. *P < 0.05 and **P < 0.01 (paired *t*-tests).

To produce a rescue of the zyxin-KO cells, the latter were stably transduced with a mouse zyxin-GFP-expressing lentivirus. Although the expression level of zyxin-GFP was low, requiring immunoprecipitation for detection (Fig. 5A), 57% of the transduced cells were GFP-positive by flow cytometry (Fig. 5B), and GFP striations in the basal layers of the cells resembled the pattern of zyxin immunofluorescence in WT RPE cells (Fig. 5C). WT, KO, and repair RPE cells were infected with HSV1-GS5451 for 6 hours to determine if HSV-1 susceptibility was decreased in the rescue cells. KO cells were infected at 40% greater proportions (*P* < 0.01) and emitted 31% more fluorescence than WT (*P* < 0.05), and this was decreased to a residual 16% in the rescue cells (Fig. 5D).

### Zyxin restricts HSV-1 and PRV infection

**Fig 6:**
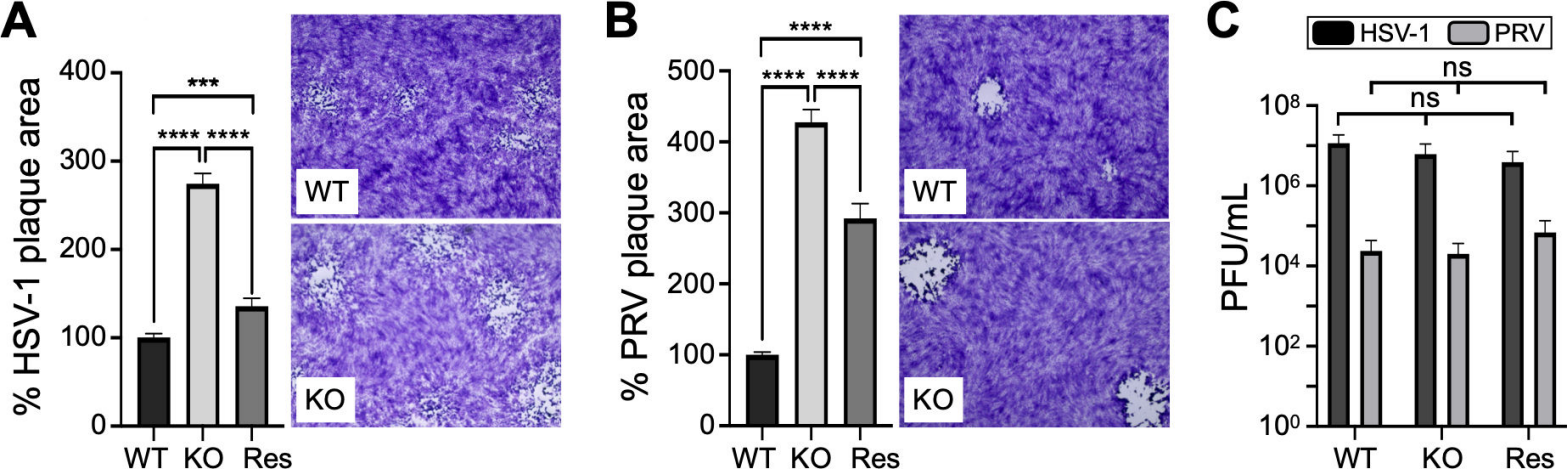
HSV-1 and PRV propagation in the absence of zyxin. (**A**) Size of HSV-1 plaques produced on wild-type, zyxin-KO (KO), and rescue (Res) RPE cells at 3 dpi. Mean plaque areas from a minimum of 40 plaques across either four (WT and KO) or two (Res) replicas are normalized to WT with standard error. Representative images of plaques are provided. (**B**) Size of PRV plaques at 3 dpi. A minimum of 70 plaques were measured across either four (WT and KO) or one (Res) replicas. (**C**) HSV-1 and PRV titers (PFU/mL) produced from indicated cells at 24 hpi. Standard error is shown for each sample. Not significant (ns; *P* > 0.05), ***P < 0.001, and ****P < 0.0001 (multiple *t*-tests Holm-Šidák correction).

Similar to the HSV-1 single-round reporter, infection with replication-competent HSV-1 and PRV was attenuated by zyxin (Fig. S5D). This effect became more pronounced over multiple rounds of infection based on plaque growth over a 3-day period (*P* < 0.0001) (Fig. 6A and B). However, the increase in plaque size on the KO cells was not associated with a corresponding increase in virion production (Fig. 6C). Collectively, these findings support a role for zyxin in restricting alphaherpesvirus infection at an initial step of infection.

## DISCUSSION

Mammalian alphaherpesviruses, including HSV-1 and PRV, are neuroinvasive pathogens that establish life-long latent infections in the peripheral nervous system. Following fusion into a cell, these viruses deliver their DNA-containing capsids to nuclei by a two-step intracellular transport mechanism. The capsid first engages the dynein microtubule motor to traffic to the centrosome, which in neurons is responsible for long-distance retrograde axonal transport into the nervous system (19). Once at the centrosome, conventional kinesin-1 routes the capsid to the nucleus (20). This mechanism is directed by two essential tegument proteins, pUL36 and pUL37, that remain attached to the capsid surface as components of the post-entry capsid complex (7–9).

In this study, HeLa cell proteins in close proximity to the PRV PECC were identified by biotin proximity labeling. Three PRV recombinants incorporating BioID2 into the virion architecture, as fusions to pUL36 or pUL37, were produced for this portion of the study. These viruses were designed to produce overlapping results to help validate the approach and broaden the scope of the analysis. Two BioID2 fusions to pUL36, at either end of the protein, were included to improve the identification of proximal proteins due to the size of the protein (>300 kDa). Small perturbations in pUL36 and pUL37 can have significant impacts on viral propagation, and the finding that the BioID2 fusions to these proteins were well tolerated by PRV indicated that protein function was not substantially altered. While B36 suffered a mild defect in propagation, incorporation, and expression, we kept it in our study both to maximize host target identification and to reduce the detection of false positives (a strategy that has been implemented by other proximity-labeling studies) (40).

Proximity labeling could initiate immediately upon viral entry into cells because BioID2 was incorporated into the virion architecture. While the approach allows for the detection of substrates that the PECC may encounter either transiently, indirectly, or weakly during initial infection, a technical limitation of BioID2 is its slow biotinylation kinetics. We determined that an 18-hour infection was necessary to obtain sufficient substrate labeling for robust mass spectrometry detection. For context, HSV-1 and PRV arrive at nuclei within 1 hour of infection (41), and transcription of UL36 and UL37 occurs at 3–12 hpi (42, 43). Therefore, the experimental design is expected to produce biotinylated proteins from incoming PECCs as well as during the later assembly/egress phase of infection when pUL36 and pUL37 are expressed *de novo* (Fig. S1). Although our intent was to examine PECC interactions during initial infection, we opted against chemically inhibiting protein expression (e.g., through the addition of cycloheximide) in favor of preserving cell physiology. TurboID variants of BioID2 could be effective for future studies but were not available when the pilot studies for this project were initiated (44). Instead, this technical limitation was addressed by the inclusion of a follow-up CRISPR screen to specifically examine phenotypes during initial infection (see below).

The BioID2 strategy identified both viral and cellular proteins. While the former were not the focus of this study, they merit some discussion. B36/36B identified itself and pUL37 as top candidates, and 37B reciprocally identified pUL36 and itself (Fig. S2). To a lesser extent, 36B and 37B also identified pUS3, which is a serine-threonine kinase and a PECC component (8, 45). Although the capsid protein to which pUL36 directly binds, pUL25, was not identified, the major capsid protein, VP5, was one of the more significant viral protein hits. Capsid triplex protein VP19c was also detected, and there is an indication that pUL36 may interact with the capsid triplex in the betaherpesvirus, HCMV (46). The absence of pUL25 in the BioID2 data set is unlikely an indication that the well-established PECC pUL36-pUL25 interaction was absent during infection (47–50), but rather that lysine residues in pUL25 were presumably inaccessible to the BioID2 enzyme (51). Other structural components of the virion identified included three tegument proteins: pUL21, pUL46 (VP11/12), and pUL51. Of these, pUL46 is not recognized as a component of the HSV-1 or PRV PECC (9), while pUL21 is a candidate PECC component (52, 53). Whether pUL51 is a PECC component has not been investigated to our knowledge, but interestingly the protein localizes to cellular focal adhesions in proximity to zyxin (54).

By far the strongest cellular proximity factor identified was RANBP2/NUP358, a cytoplasmic-facing nuclear pore protein that, along with pUL36, is implicated in HSV-1 nuclear docking (55). Since capsids remain docked at the nucleus after the DNA genome is released, it would reason that BioID2 has an extended opportunity to biotinylate NUP358. NUP214, another nuclear pore protein implicated in HSV-1 nuclear docking and genome release, was also detected (55–57). Other cellular proteins that are reported to support HSV-1 infection include HCF-1 (HCFC1) (58, 59) and DDX3X (59–62). DDX3X was previously reported as a component of mature HSV-1 virions (63), interacts with the PECC component pUS3, and is required for efficient packaging of pUS3 into mature particles (60). It is therefore possible that proteins such as DDX3X may reside within the labeling radius of 37B, 36B, and B36 viruses due to its interaction with pUS3, which is tethered to the capsid by pUL36 (17). Several microtubule-associated proteins (MAPs) were detected including MAP1, MAP4, EPB41L2, Jupiter-homolog (HN1), the dynein adaptor BicD2, and kinesin-8 (KIF18B). ZO-1 (TJP1) is a microtubule-associated cell junction protein that modulates infections of several RNA viruses (64), but the loss of which is implicated in enhancing cell-cell spread of HSV-1 (65). The centrosome is a pivotal junction for PECC trafficking, and centrosomal proteins were enriched in the 37B proximity hits, including CEP85, CEP170, CEP350, HAUS6, and HAUS8 (14, 20, 41, 66).

Despite the compelling nature of the BioID2 results, proximity labeling alone does not inform significance (24, 26, 29, 67, 68). To assess the functional relevance of the BioID2 data sets, a subset of the cellular proteins that were identified by all three PRV BioID2 viruses was tested in the arrayed CRISPR screen. Furthermore, to enrich for proteins that are fundamental to the conserved intracellular trafficking mechanism used by PRV and HSV-1, we developed an HSV-1 single-round reporter virus to assess cellular function during initial infection. The screen used transient delivery of CRISPR-Cas9 RNPs without selection, which can reduce off-target effects due to the transient nature of the Cas9 activity. Knock-out efficiency was not tested across all targets in the screen, so genes that did not yield a significant change in infection may not have been successfully reduced in their expression. The “proviral” targets that resulted in decreased infection upon KO contained proteins that are consistent with existing literature, including Ran and PUM1 (37, 38). RAN is critical for HSV-1 capsid docking at nuclear pores (37) and knockdown reduces HSV-1 replication (59). PUM1 promotes HSV-1 infection by suppressing LGP2, a promoter of innate immunity genes (38). Targeting the proteasomal component PSMC2 (also known as Mss1) resulted in the strongest decrease in reporter activity, but the role of PSMC2 during HSV-1 infection is controversial (39, 69). Our attempts to recover clonal PSMC2 KO isolates were unsuccessful, potentially due to the observed decrease in cell viability.

The WASH (Wiskott–Aldrich syndrome protein and SCAR homolog) complex proteins strumpellin (KIAA0196) and FAM21C emerged as effectors that, when targeted by CRISPR, resulted in increased early HSV-1 infection, an effect that was not previously described. The WASH complex is involved in endosomal sorting, with strumpellin and FAM21C regulating Arp2/3-mediated actin branching (70). Full knockouts of either target could not be isolated from the transient CRISPR library or by targeting with lentivirus. KO of strumpellin is embryonic lethal in mice (71) and inhibits proliferation in amoeba (72). Despite the lack of KO cells, cells with reduced levels of either protein were validated as being more susceptible to HSV-1. Curiously, strumpellin and FAM21C were reported as components of purified HSV-1 virions of unknown significance (73, 74).

CRISPR-Cas9 targeting of zyxin produced one of the strongest changes in HSV-1 early infection in our screen. Clonal zyxin knockout cells were hyper-susceptible to HSV-1 based on reporter gene expression (HSV1-GS5451) and HSV-1 and PRV based on fluorescent capsid expression (HSV1-GS6807 and PRV-GS1846). Rescue of zyxin-KO cells with even a modest amount of murine zyxin significantly diminished HSV-1 reporter gene expression. The most significant impact of zyxin knockout was observed in plaque size, which unlike the reporter gene expression assays occurs over multiple rounds of infection. HSV-1 and PRV plaque sizes were substantially increased in the absence of zyxin while virus titers remained unchanged. This is consistent with zyxin restricting an early step of infection (e.g., by slowing genome delivery) but not subsequently impairing virus production thereafter. There are no roles for zyxin ascribed to herpesvirus infection and several hypotheses could explain the “antiviral” phenotype observed in this study. The canonical role for zyxin is that of a mechano-sensitive component of focal adhesions that rapidly localizes to actin filaments in response to mechanical stressors, promoting the stability of the actin cytoskeleton (75, 76). Zyxin also interacts with nectins (77). Nectin-1 is the receptor for HSV-1 in many cells including RPE cells (34, 78). Although it is unclear what role zyxin has on nectin-1, zyxin could mediate the surface availability of nectin-1. Alternatively, cell junctions and cortical actin are barriers to infection that may be disrupted in the absence of zyxin (79). The PECC pUS3 kinase promotes breaching of the cortical actin, and it is possible that its mode of action includes regulating zyxin (10, 80). There are other reports of zyxin functioning outside its traditional cytoskeletal roles. Zyxin can also shuttle to the nucleus (81), regulate the transcription of various genes (82), and regulate the stability of specific mRNA (83). Human papillomavirus infection increases zyxin nuclear translocation, possibly through the E6 protein (84). Recently, zyxin was demonstrated to promote type I IFN responses by mediating the interaction between mitochondrial-activated signaling protein and RIG-I-like receptors. Zyxin knockdown reduced the extent of IFN production in response to influenza A or poly:IC treatment (85).

Biotin proximity labeling has been used in multiple studies to investigate host-pathogen interactions (24, 40, 67, 86–90). Our approach to structurally incorporate a BioID probe into a virus, as opposed to overexpression in transfected cells, was previously achieved with HIV and HCMV (28, 29). Although equivalent studies for alphaherpesviruses were not previously reported, a related HSV-1 study by Griffiths and colleagues (59) combined a yeast-2-hybrid screen with a siRNA knockdown screen in HeLa cells that was coupled with a reporter virus at a late time point (1–3 dpi). Given the differences in approach, it may not be surprising that the overlap in the identified cellular proteins between our study and those by Griffiths et al. (59) was limited. Griffiths et al. (59) did not identify cellular partners for pUL37, and the three reported for pUL36 (HGS, KRTAP4-12, and CCNDBP1) were not identified here. Nevertheless, five proteins identified as significant by Griffiths et al. in the siRNA screen were also detected by all three PRV-BioID2 in the current study: HCFC1, RAN, dishevelled 1 (DVL1), inhibitor of K562 cytokine (IK), and an H2B histone (HIST2H2BE). DVL1 was one of several proteins identified in our proximity screen that participates in WNT signaling.

Proximity-labeling approaches have proved advantageous in identifying relevant host targets within the proximity “cloud” that may be overlooked with other biochemical approaches. However, the breadth of target capture achieved through this approach necessitates an orthogonal method to identify targets with biological significance. The CRISPR screen revealed proviral and antiviral cellular proteins including zyxin, while also providing validation for the broader BioID2 data sets. Uncovering the mechanisms by which herpesviruses manipulate or circumvent cellular factors during the initial stage of infection should aid in the development of non-neuroinvasive prophylactic vaccines and better host-directed therapies for acute infection to improve patient outcomes.

## MATERIALS AND METHODS

### Cells

All cells were cultured in Dulbecco’s modified Eagle medium (DMEM) with either 10% BGS (PK15 and Vero) or 10% FBS (Vero 8-1, HeLa, and hTERT-RPE1). Vero 8-1 cells were a gift from Dr. Fred Homa (University of Pittsburg School of Medicine). A derivative of the hTERT-RPE1 cells knocked out for zyxin was produced as part of this study, as was a derivative of the latter that was stably transduced to express mouse zyxin translationally fused to GFP. For all hTERT-RPE1 cells, the medium was supplemented with 1 mM sodium pyruvate, and 4 µg/mL blasticidin was also added for the zyxin-GFP transduced derivatives. All cells were maintained at 37°C in 5% CO_2_, kept below passage 35, and routinely checked for mycoplasma contamination.

### Viruses

Recombinants of HSV-1 strain F and PRV strain Becker were produced using previously described infectious clones and two-step RED-mediated recombination (91–94). Three recombinants of PRV that express BioID2 as a fusion to either pUL36 or pUL37 were produced for this study. A PCR template, pEP-BioID2-in, was first derived from pcDNA3.1, encoding the 232 aa BioID2 coding sequence (gift from Dr. Benjamin Benson). Briefly, a portion of the BioID2 open reading frame was duplicated, and the aphAI gene (encoding resistance to kanamycin) and an I-sceI cleavage site were inserted between the duplicated sequences, resulting in pGS6585 (aka, pEP-BioID2-in). Linear DNA products were produced from pEP-BioID2-in by PCR and RED-recombined into a previously described PRV clone, pGS5298, that encodes a GFP-5xG-UL26 internal capsid tag (95). This resulted in pGS6590 [aka “B36”; pGS5298 + BioID2 UL36 (double in-frame insertion between UL36 codons 1 and 2)], pGS6591 [aka “37B”; pGS5298 + UL37-BioID2 (double in-frame insertion between UL37 codons 918 and 919)], and pGS6627 [aka “36B”; pGS5298 + UL36-BioID2 (double in-frame insertion between UL36 codons 3904 and 3905)]. Transfections of these infectious clones into PK15 cells produced infectious viruses (PRV-GS6590, PRV-GS6591, and PRV-GS6627) that were propagated an additional round and stored as aliquots at −80°C, as described previously (7). For proximity labeling experiments, PRV stocks were expanded in three 15 cm dishes of PK15 cells that were grown to 80%–90% confluency and infected at MOI:10. The supernatants were harvested into two 50 mL conicals on ice when all cells exhibited complete cytopathic effect (24–48 hpi). The supernatants were centrifuged for 20 min at 5,000 × *g* at 4°C to remove cellular debris, transferred to an ultracentrifuge tube (Seton #7052), underlaid with a 20% (wt/vol) sucrose in PBS, and centrifuged at 20,764 × *g* (13,000 rpm) for 1 hour in a SW32 rotor at 4°C. The pellet was resuspended in 500 µL of PBS on ice, and 50 µL aliquots were stored at −80°C. This procedure minimized the contamination of the PRV stocks with biotinylated proteins from the producer cells.

The HSV-1 single-round immediate-early gene reporter, HSV1-GS5451, was derived from the previously described HSV1-GS3217 that encodes a CMVIE > NLS-tdTomato expression cassette inserted in the US5 gene (96). HSV1-GS5451 carries a deletion in the UL25 gene that prevents the production of DNA-containing viral particles and was propagated on Vero 8-1 cells that express pUL25. Supernatants were harvested, sonicated at 1.5 s on and 1 s off for 10 cycles, centrifuged at 300 × *g* for 5 min to remove cellular debris, and single-use aliquots were stored at −80°C. Titers were determined by plaque assay on standard Vero cells and Vero 8-1 cells in parallel. HSV1-GS5451 stocks consistently did not produce plaques on standard Vero cells (titer < 5 PFU/mL), validating replication incompetency. Replication-competent viruses used to assess the infectivity of cells by flow cytometry included PRV-GS1846 (pBecker3 + mRFP1 UL35) and HSV1-GS6807 (pHSV-F-CREin + UL25/mCherry), both previously described (97–99).

Prior to use, aliquots of all virus stocks were thawed on ice and sonicated in a VCX-500 cup horn ultrasonic processor (Sonics and Materials, Newton, CT, USA) for 10 cycles: 1.5 s on and 1 s off.

### Plaque assays

Plaque assays were used to determine viral titer and as a metric of cell-cell spread (plaque size). Infections were performed on confluent cells in 6-well plates. PK15 cells were used to measure PRV titers and diameters, and WT Vero cells and Vero 8-1 cells were used to measure HSV-1 titers. Viral stocks were serially diluted to determine titers (PFU/mL), and plaque diameters were measured at 24–48 hpi as described previously (20). Plaque area measurements and titers using RPE cells were performed as previously described (100–102). Briefly, WT, zyxin-KO, and repair RPE cells were grown to equal confluency on 6-well plates and infected with 100 PFU of either HSV1-GS3217 or WT PRV (provided by Dr. Tamar Ben-Porat) for plaque area measurements. Infected cells were Giemsa-stained at 3 dpi and imaged by transmitted light microscopy on the EVOS Cell Imaging System (Thermo Fisher Scientific). Plaque area was determined using the radius of at least 30 randomly selected plaques per condition. Titers produced on RPE cells were determined after infecting with HSV1-GS3217 at MOI:0.01 or WT PRV at MOI:0.1 for 24 hpi. Harvested samples were subjected to three freeze-thaw cycles prior to titration on Vero cells.

### Preparation of H-banded virions

Per virus, six 15 cm dishes of PK15 cells were infected at MOI:10 for 24 hours. Infected supernatants were centrifuged at 5,000 × *g* for 20 min at 4°C to remove cellular debris, underlaid with a 10% Nycodenz (Fisher Sci #AN1002423) in PBS solution, and centrifuged at 20,764 × *g* (13,000 rpm) in a SW32 rotor for 60 min at 4°C. Viral pellets were resuspended in PBS and overlayed on a 12%–32% continuous dextran T10 (Pharmacosmos A/S #5510-0010-4007) gradient that was prepared in PBS the day before on a BioComp Gradient maker Pro set at an angle of 81.5°, 20 rpm, 90 s. The samples were centrifuged in a SW41 rotor at 49,370 × *g* (20,000 rpm) for 60 min at 4°C. Heavy (H) particles were visualized and extracted through the tube wall using a 21-gauge needle and syringe. The H-particles were pelleted out of dextran at 37,408 × *g* (20,000 rpm) for 60 min at 4°C in a SW50.1 rotor, resuspended in 60 µL 4× final sample buffer [8% SDS, 40% glycerol, 0.25 M Tris (pH 6.8), and 0.02% bromophenol blue], and frozen at −20°C. Prior to visualizing on a protein gel, samples thawed on ice were supplemented with 50 mM DTT and boiled for 5 min.

### Western blot analysis

Protein lysates were produced by adding 2× final sample buffer supplemented with a final concentration of 50 mM DTT directly to cells (either *in situ* or following pelleting) and boiled for 5–10 min. Samples were run on a 4%–12% SDS gel and transferred to a 0.45 µm Immobilon FL PVDF membrane (ThermoFisher Scientific #IPFL00010). When detecting biotinylated proteins, 5% BSA in PBST (PBS + 1% Tween 20) was used for blocking, and 1% BSA in PBST was used to dilute IRDye 680RD Streptavidin (LiCor #926-68079) at 1:1,000 and for wash steps. For all antibodies, blocking was done in 5% milk in PBST, and antibody incubations and washes were done in 1% milk in PBST. Anti-BioID2 antibody (Abcam #232733) was used at 1:1,000, anti-pUL36 9k (rabbit polyclonal generated by immunization with PRV pUL36 peptide sequence HTVGGRPSRKFRPR) was used at 1:3,000, anti-pUL37 D1789 (rabbit polyclonal generated by immunization with PRV pUL37 peptide sequence REAADRVLGDYHE) was used at 1:2,500, anti-VP5 [mouse clone 3C10 (gift from Dr. Lynn Enquist) was used at 1:2,000], anti-tubulin (Dm1A #ab7291) was used at 1:10,000, anti-zyxin (Invitrogen #PA1-25162, Millipore Sigma #Z0377) was used at 1:1,000 and 1:2,000, respectively, anti-strumpellin (Santa Cruz #B-10 #sc-377146) was used at 1:1,000, anti-FAM21C (Millipore Sigma #ABT79) was used at 2.5 µg/mL, and anti-GFP antibody (Invitrogen #A6455) was used at 1:5,000. All fluorescent secondary antibodies were used at 1:10,000, and m-IgG1-BP-HRP (Santa Cruz #sc-525415) was used at 1:2,500.

### Proximity labeling and sample preparation

HeLa cells were grown in six 15 cm dishes to 40%–50% confluency, biotin was added to a final concentration of 20 µM, and the cells were incubated for another 16–24 hours until 100% confluency. The cells were infected with sucrose-purified PRV at MOI:3 for 18 hours in DMEM maintained with 20 µM biotin and 2% FBS. The cells were placed on ice, washed with cold PBS three times, and lysed with RIPA buffer [50 mM Tris-Cl (pH 7.5), 250 mM NaCl, 1 mM EDTA, 1% NP40 (vol/vol), 0.1% SDS, and 0.5% sodium deoxycholate] containing Sigma Protease Inhibitor (Sigma Aldrich P8340) and 1 mM DTT for 1–2 hours at 4°C. The lysates were cup horn sonicated for 30 cycles of 1.5 s on and 1 s off and centrifuged at 12,000 × *g* for 20 min at 4°C to pellet insoluble material. Supernatants were incubated with streptavidin-conjugated agarose beads (Pierce #20347) on a rotator at 4°C for 16–24 hours. Protein-bead conjugates were processed in the following solutions: twice in 2% SDS, once in 50 mM HEPES (pH 7.6), 1 mM EDTA, 0.1% deoxycholate, 1% Tx100, once in 10 mM Tris-Cl (pH 7.4), 250 mM LiCl, 1 mM EDTA, 0.1% deoxycholate, 1% NP-40, once in 2 M urea, and finally in 50 mM Tris (pH 7.4) before protein elution (103). For mass spectrometry, the protein samples were precipitated using the chloroform/methanol method, denatured with 8 M urea in 50 mM ammonium bicarbonate (ABC), vortexed 1 hour at RT, and processed with ProteaseMAX according to the manufacturer’s protocol (Promega). Samples were reduced with 5 mM Tris(2-carboxyethyl)phosphine (TCEP), vortexed for 1 hour at RT, alkylated in the dark with 10 mM iodoacetamide for 20 min, diluted with 50 mM ABC, and quenched with 25 mM TCEP. Samples were digested with sequencing grade trypsin overnight at 37°C with shaking, centrifuged at 15,000 × *g* for 15 min at RT, transferred to a new tube, and acidified with trifluoroacetic acid to a final concentration of 0.1%. A total of 25 µg of each sample was desalted with Pierce C18 spin columns (Thermo Fisher Scientific, #89873) per the manufacturer’s instructions and dried by vacuum centrifugation prior to MS analysis (104).

### Analysis by LC-MS/MS

Dried samples were resuspended in 20 µL of buffer A (94.875% H_2_O with 5% ACN and 0.125% FA), and 3 µg, as determined by microBCA assay (23235; Thermo Fisher Scientific), of each fraction or sample was loaded via autosampler with UltiMate 3000 HPLC pump onto a vented Pepmap 100, 75 µm × 2 cm, nanoViper trap column coupled to a nanoViper analytical column (Thermo Fisher Scientific) with a stainless steel emitter tip assembled on the Nanospray Flex Ion Source with a spray voltage of 2,000 V. A coupled Orbitrap Fusion was used to generate MS data. Buffer A contained 94.785% H_2_O with 5% ACN and 0.125% FA, and buffer B contained 99.875% ACN with 0.125% FA.

Protein identification/quantification and analysis were performed with the Integrated Proteomics Pipeline-IP2 (Integrated Proteomics Applications) using ProLuCID (105, 106), DTASelect2 (107, 108), Census, and QuantCompare. Spectral raw files were extracted into MS1 and MS2 files (and MS3, when appropriate) using RawConverter v1.0.0.0. The tandem mass spectra were searched against a combined human and suid herpesvirus protein database downloaded on 30 May 2017. Searched spectra were matched to sequences using the ProLuCID/SEQUEST algorithm (ProLuCID v3.1) with 50 ppm peptide mass tolerance for precursor ions and 600 ppm for fragment ions. ProLuCID searches included all fully and half tryptic peptide candidates that fell within the mass tolerance window and had unlimited miscleavages. Carbamidomethylation (+57.02146 D) of cysteine was considered a static modification. Only peptides with 10 PPM of expected *m/z* were considered, and we considered proteins identified by one or more peptides.

### Gene annotation

Metascape (33) (http://metascape.org) was used to conduct a functional enrichment analysis using the GO Biological Processes and GO Cellular Components input gene lists and the following pathway and process enrichment parameters: minimum overlap (3), *P*-value cutoff (0.01), and minimum enrichment (1.5). For the simplified visualization in Fig. 2B, we removed the GO terms encompassing host targets that were entirely or largely encased within other GO categories that were more statistically significant. GO:1903311 (regulation of mRNA metabolic process, specified as “mRNA regulation”) encases GO:1903313 and GO:0061013. GO:0005643 (nuclear pore) contains proteins largely overlapping with GO:0050657 and GO:0016363. Finally, GO:0030055 (cell-substrate junction) largely encompasses host targets from GO:0005912, GO:0097435, and GO:0002102.

### Functional network map

STRING version 11.5 (109, 110) (https://version-11–5.string-db.org/) was used to generate the basic physical network map using the following parameters: network type (physical subnetwork), network edge (confidence), active interaction sources (experiments/databases), and minimum required interaction score (0.4/medium/query proteins only). The thickness of the edges (linkage lines between target nodes) indicates the confidence of the experimentally and database-obtained data supporting the protein-protein interaction. Target nodes are shaded according to the strength of spectral count determined by log_2_ (average spectral count across the three PRV BioID2 infections). Seven categories were associated to the target nodes: mRNA regulation (GO:1903311, GO:1903313, and GO:0061013), nuclear pore; nuclear speck (GO:0005643 and GO:0016607), cell-substrate junction, actin-cytoskeletal organization (GO:0030055, GO:0005912, GO:0097435, and GO:0002102), translation initiation (GO:0006446), neuron arborization (GO:0150012), WASH complex (GO:0071203), and immune/viral response (GO:0039531 and GO:0045087).

### Microscopy-based gene reporter assays

Cells were seeded at 2 × 10^5^ per well of a 6-well tray containing a flame-sterilized glass coverslip. After 16–24 hours, cells were infected with untagged HSV-1 strain F (HSV1-GS2695) or the single-round immediate early reporter virus (HSV1-GS5451) at the noted MOIs and times. At 1 hpi, the inoculum was removed and replaced with DMEM supplemented with 2% FBS for the remainder of the infection. Hoechst nuclear stain (Sigma-Aldrich 33258) was added directly to the wells in the last 30 min of infection at 37°C. At the end of infection, cells were washed once with PBS and fixed with 4% EM-grade paraformaldehyde (PFA) for 15 min at RT. After washing three times with PBS, coverslips were mounted on glass slides with ProLong Gold Antifade Mountant with DAPI (Fisher Scientific P3693) and sealed. Coverslips were imaged using a Plan Fluor 40×/1.3 NA oil objective using 500 ms exposures for blue and red channels. A minimum of 700 nuclei were imaged per sample. A custom Image J macro was used for the automated detection of nuclei and the associated RFP intensities. Cells were scored positive for infection if nuclear integrated red fluorescence was above the maximum nuclear fluorescence measured from cells infected with a non-fluorescent HSV-1 at the matched MOI.

### CRISPR KO and functional assay

Four guide RNAs per gene were obtained and resuspended at 160 µM each (Horizon Bioscience). The four guide RNAs were pooled at equimolar concentration, for a final concentration of 40 µM each. All guide sequences are provided in File S3. TracrRNA, guideRNA, and Cas9 protein were complexed at a molar ratio of 1:1:2. TracrRNA and guideRNA were first complexed together for 30 min at 37°C, then with Cas9 for 15 min at 37°C to form CRISPR-Cas9 ribonucleoprotein complexes. CRISPR Cas9 RNPs were aliquoted and stored at −80°C until use. Per gene target, 2 × 10^6^ RPE cells were electroporated with 4 µL CRISPR-Cas9 RNPs on the Lonza 4D nucleofector with P3 buffer and protocol EA-104. Cells were recovered for 30 min at 37°C before being replica-plated into seven flat-bottom 96-well plates. After 72 hours, one replica plate was resuspended in 10% dimethyl sulfoxide (DMSO) in FBS and frozen at −80°C for cryopreservation, and the other was stained with the amine-reactive Ghost Dye Red 710 (Tonbo Biosciences) for the assessment of cell viability by flow cytometry. The remaining plates of cells were infected with HSV1-GS5451 at MOI:50 for 8 hours in triplicate. In parallel, a plate of unedited RPEs was infected for 8 hours. Infections were carried out at 37°C in 50 µL inoculum volumes and agitated every 15 min for 1 hour. After 1 hour, the infection inoculum was removed, and the wells were replaced with 2% FBS in DMEM for the remaining 7 hours. At the end of the infection, cells were lifted with 0.125% trypsin, pelleted, and washed in PBS containing 0.5% BSA and 2 mM EDTA, and resuspended in PBS containing 1% PFA. Plates were kept foil-wrapped at 4°C until run on the flow cytometer. The screen was repeated twice. Within each screen, the average log_2_-fold change in tdTomato emissions was obtained for each well (e.g., each CRISPR target) relative to the plate median (log_2_fc) across triplicate plates. *Z* scores were calculated for each KO and were aggregated using Stouffer’s method across screens. Targets that fell within the 95% threshold (*Z*: −1.96–1.96) were selected for further validation of functional significance.


$$Stouffer^{\prime }s\;Average=\frac{\sum \frac{X\;-\mu }{\sigma }}{\sqrt{2}}$$

### Flow cytometry-based infectivity assays

Cells were seeded in a 96-well flat bottom plate 16–20 hours prior to assay. Cells were infected in triplicate with the noted MOI and virus for 1 hour in a total inoculum volume of 40 µL, then replaced with 2% serum media for the remaining infection time. Viruses and MOIs used in these assays included HSV1-GS5451 MOI:30, HSV1-6807 MOI:5, and PRV-1846 MOI:30. At the noted time of harvest, cells were washed once in PBS, once in PBS containing 0.5% BSA and 2 mM EDTA, then resuspended in PBS containing 1% PFA. Plates were kept foil-wrapped at 4°C until run on the flow cytometer.

### Generation of cells targeted for KO of strumpellin and FAM21C

The LentiCRISPRv2 vector system was used to generate lentiviral vectors, each encoding a single gRNA against strumpellin or FAM21C (111, 112). Using the Brunello library optimized for minimizing off-targets and maximizing editing efficiency (113), four gRNAs and four lentiviral vectors were designed to target strumpellin (S-gRNA1 through S-gRNA4) and FAM21C (F-gRNA 1 through F-gRNA 4). Briefly, double-stranded DNA oligonucleotides corresponding to the chosen target sequence with the appropriate overhangs were cloned into the lentiCRISPRv2 vector (Addgene #52961), transformed into Stbl3 cells, and the purified vectors were confirmed by Sanger sequencing. A lentivirus encoding a scrambled gRNA, pJH397, with no target in the human genome was included as a non-targeting control. Each lentivirus was packaged individually in HEK293T cells transfected with 5 µg pLenti-Zyxin-eGFP plasmid, 3.3 µg p∆-NRF gag-pol-tat-rev packing plasmid, and 1.6 µg pVSV-G plasmid. Supernatants were harvested at 48 hours, passed through a 0.45 µm filter, and stored at 4°C for short-term use or −80°C. WT RPE cells were seeded at a density of 1.2 × 10^5^ cells per well of a 6-well tray and were transduced 24 hours later with single lentiviruses or a pooled 1:1:1:1 mixture of the four lentiviruses per target. The cells were split into media supplemented with 8 µg/mL puromycin 48 hours post-transduction. A non-transduced control was included to ensure the lack of cell expansion following puromycin addition. Western blot was used to assess protein depletion of the puromycin-resistant transduced cells.

### Isolating a monoclonal zyxin knockout from the CRISPR screen

In the pipeline for conducting the CRISPR KO and IE-genome assay above, a post-nucleofection replica plate was preserved in 10% DMSO in FBS at −80°C. We recovered and expanded the polyclonal zyxin-KO pool (over 12 days) and serially diluted it across a 96-well plate. Over the next several weeks, wells were monitored for clonal population outgrowth. Wells that were identified to have originated from a single cell were expanded and screened for protein depletion by immunoblot.

### GFP pulldowns

WT RPE, zyxin-KO, or zyxin-KO + zyxin eGFP (rescue) cells were lysed in RIPA buffer [10 mM Tris-Cl (pH 7.5), 150 mM NaCl, 0.5 mM EDTA, 1% Tx100, 0.1% SDS, 1% sodium deoxycholate, and 2.5 mM Mg_2_Cl] supplemented with Sigma Protease inhibitor cocktail (Sigma Aldrich 8340) and 1 mM PMSF for 30–45 min at 4°C rotating. The samples underwent 20 cycles of cup horn sonication at 1.5 s on and 1 s off and were rotated at 4°C for another 30 min (note: we have found that non-ionic detergents such as NP40 may not be sufficient to solubilize zyxin protein). Wash steps were performed with 10 mM Tris (pH 7.5), 150 mM NaCl, 0.5 mM EDTA, and 0.05% NP40. The insoluble fraction was pelleted by centrifugation at 13,000 × *g*. Soluble GFP was captured with ChromoTek GFP-Trap Agarose beads (Proteintech #gta). All input and bead-enriched samples were extracted in 2× final sample buffer supplemented with 50 mM DTT and boiled for 5–10 min prior to electrophoresis.

### Generating the zyxin-eGFP rescue cell line

The pLenti-Zyxin-Full Length (aa1-564)-EGFP plasmid was a gift from Dr. Mary Beckerle (Addgene plasmid #187692). The zyxin-eGFP lentivirus was packaged in HEK293T cells transfected with 5 µg pLenti-Zyxin-eGFP plasmid, 3.3 µg p∆-NRF gag-pol-tat-rev packing plasmid, and 1.6 µg pVSV-G plasmid. Supernatants were harvested at 48 hours, passed through a 0.45 µm filter, and stored at 4°C for short-term use or at −80°C. Zyxin-KO RPE cells seeded at a density of 1.2 × 10^5^ cells per well of a 6-well tray were transduced with the zyxin-eGFP lentivirus 1 day post-plating. The cells were split into media supplemented with 4 µg/mL blasticidin 72 hours post-transduction. A non-transduced control was included to ensure complete cell death following blasticidin addition. Blasticidin working concentration was determined by the minimum concentration required to kill non-transduced cells within 48 hours.

### Immunofluorescence

Cells were passaged through phenol red-free media three times over 10 days and seeded at 2 × 10^5^ on flame-sterilized coverslips in a 6-well tray for 16–24 hours prior to imaging. Cells were washed once with PBS, fixed with 4% PFA for 15 min, and washed thrice more in PBS. The samples were then blocked and permeabilized in PBS containing 10% FBS and 0.25% saponin for 2 hours at RT. Anti-zyxin antibody (Invitrogen PA1-25162) was diluted 1:200 in PBS containing 10% FBS and 0.025% saponin. Samples were aspirated, and the antibody solution was added and rocked at 4°C overnight in the dark. After washing thrice in PBS containing 10% FBS and 0.025% saponin, the samples were incubated with goat anti-Rabbit IgG (H + L) Cross-Adsorbed, Alexa Fluor (Invitrogen #A11011) at 1:200 for 1 hour at RT. After washing four times with PBS, the coverslips were mounted on glass slides with ProLong Gold Antifade Mountant with DAPI (Fisher Scientific P3693) and sealed. The basal layer of cells was imaged by spinning-disc confocal microscopy on a Plan Apo λ 100×/1.45 NA oil objective using 100 ms exposures.

### Flow cytometry

A minimum of 3,000 events were collected per well using the Attune NxT Acoustic Focusing flow cytometer (ThermoFisher) and Attune NxT Software. All data were exported as FCS files and analyzed using FlowJo v10.8.1. GhostRed signal (channel RL-2) was used to determine the percentage of cells that were non-viable. The gating strategy for the assessment of percent infection is outlined in Fig. S6. Briefly, cells were gated (Q2) to exclude debris and doublets. The VL-1 channel was used to detect and exclude autofluorescent cells. Only cells that were above the threshold for tdTomato (channel YL-1) determined by the untagged infection control were counted as infected. The FlowJo software was used to determine the MFI of the red-fluorescent population.

### Statistical analyses

Graphical illustrations and statistical analyses were generated using GraphPad Prism v9.4.1.

## SUPPLEMENTAL MATERIAL

File S1 mbio.01445-24-s0001.xlsx Cellular proximity hits.

File S2 mbio.01445-24-s0002.xlsx Viral proximity hits.

File S3 mbio.01445-24-s0003.xlsx CRISPR data.

Supplemental Figures mbio.01445-24-s0004.pdf Figures S1 to S6.

Legends mbio.01445-24-s0005.pdf Legends for Figures S1-S6.

ASM does not own the copyrights to Supplemental Material that may be linked to, or accessed through, an article. The authors have granted ASM a non-exclusive, world-wide license to publish the Supplemental Material files. Please contact the corresponding author directly for reuse.
