# Supplementary figures and images for: Leveraging biotin-based proximity labeling to identify cellular factors governing early alphaherpesvirus infection
Source: mBio. 2024 Jul 2;15(8):e01445-24. doi: 10.1128/mbio.01445-24 (PMC11323796; doi:10.1128/mbio.01445-24)

Figure S1

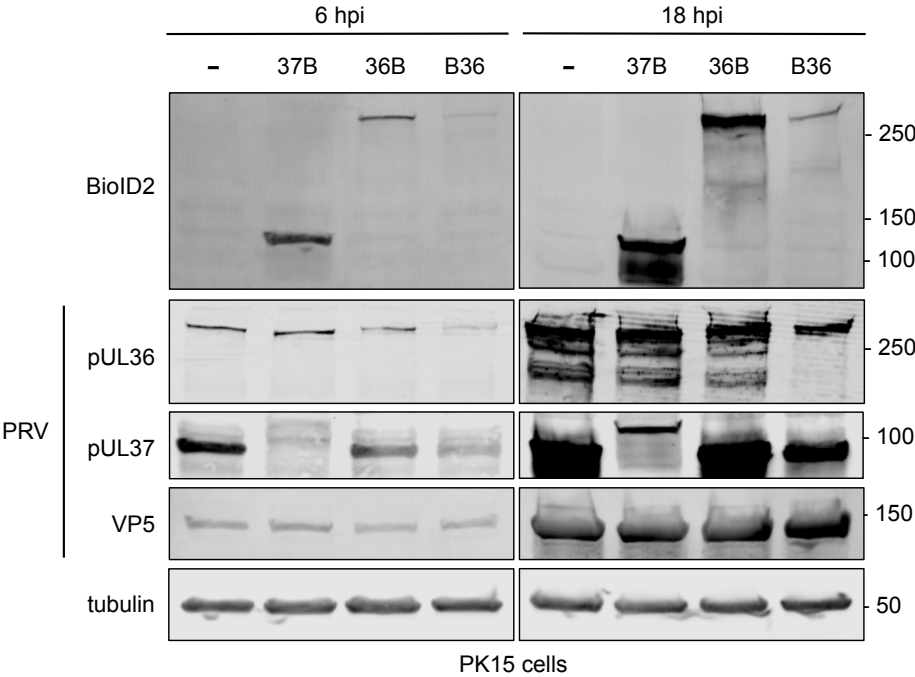

Figure S2

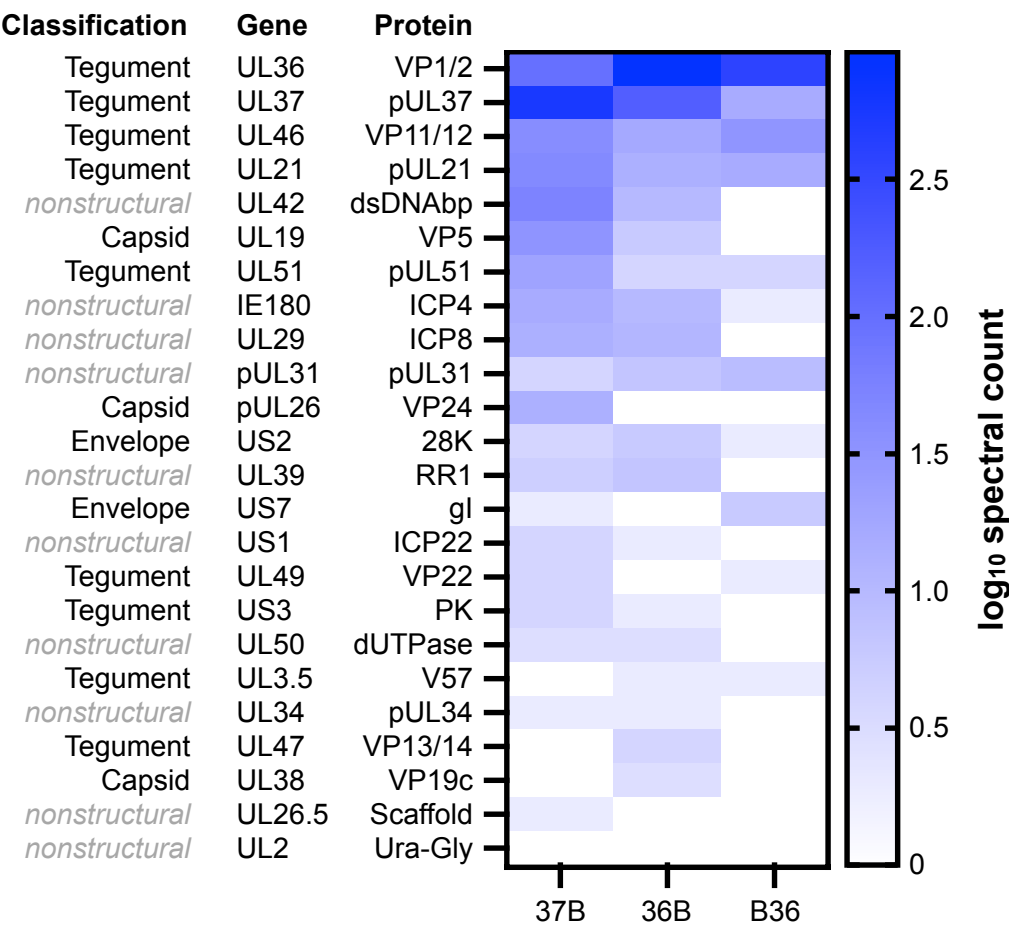

Figure S3

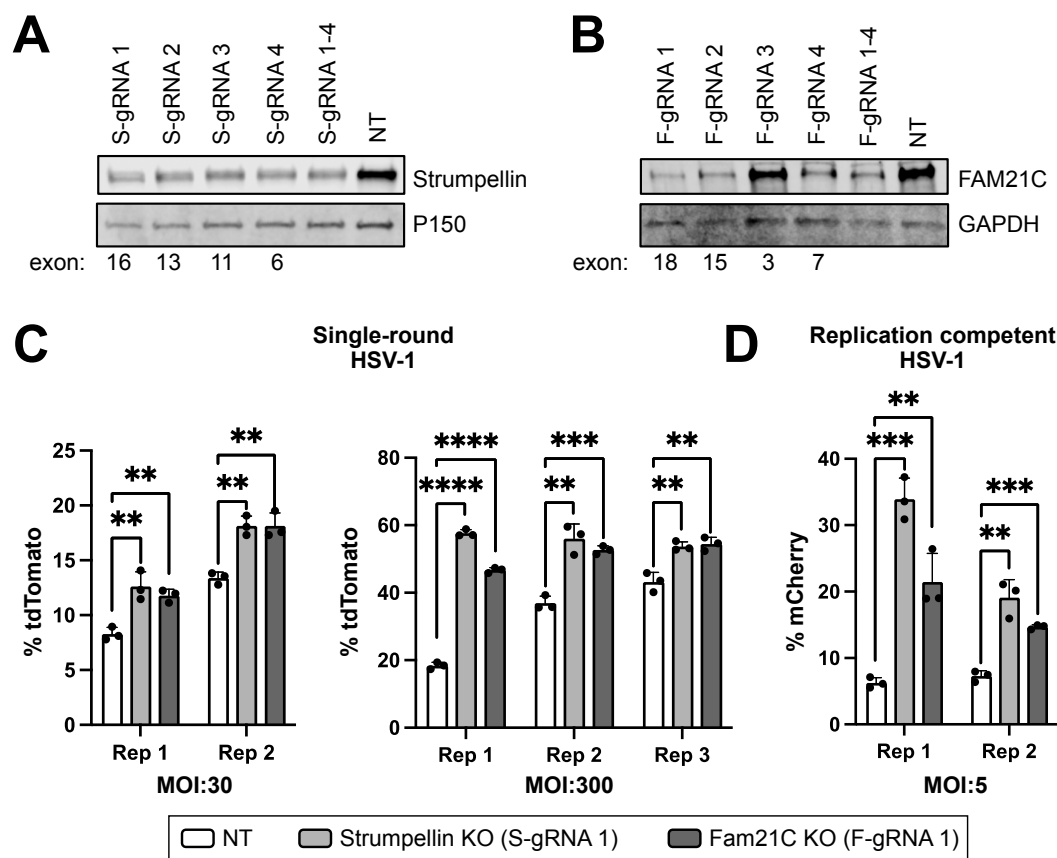

Figure S4

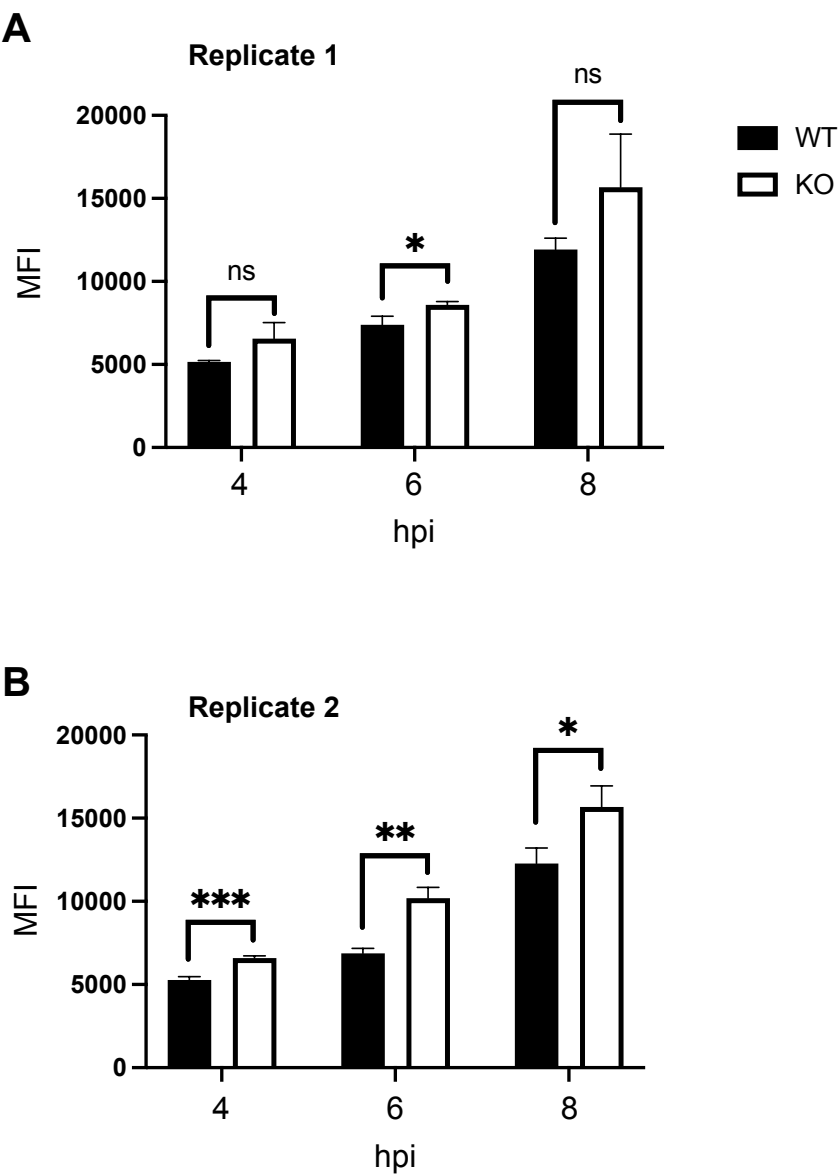

Figure S5

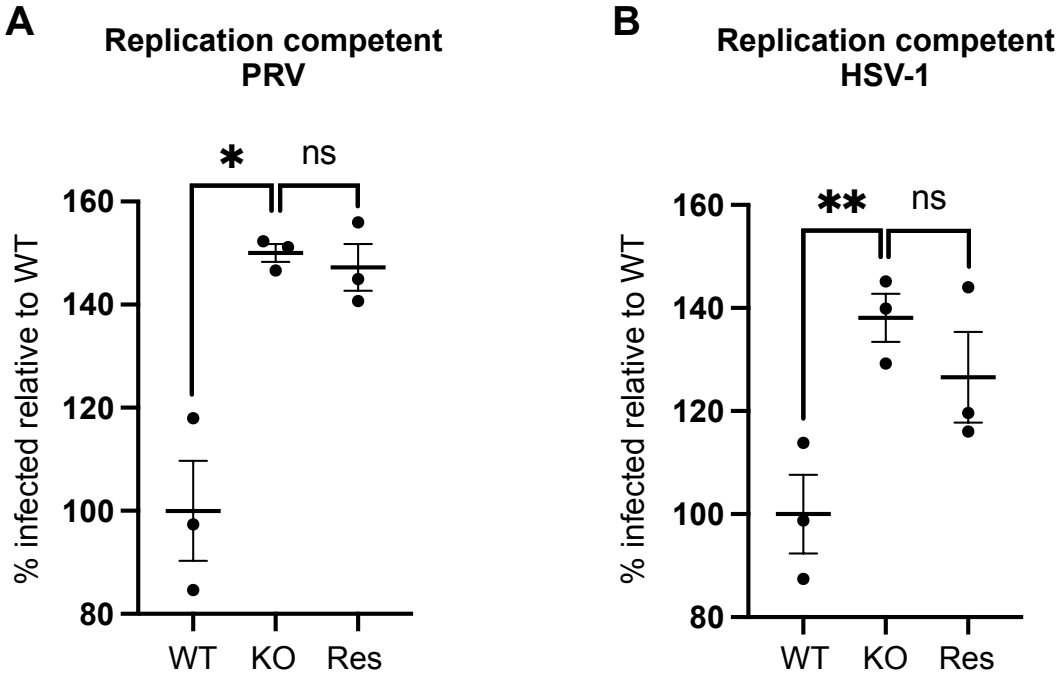

Figure S6

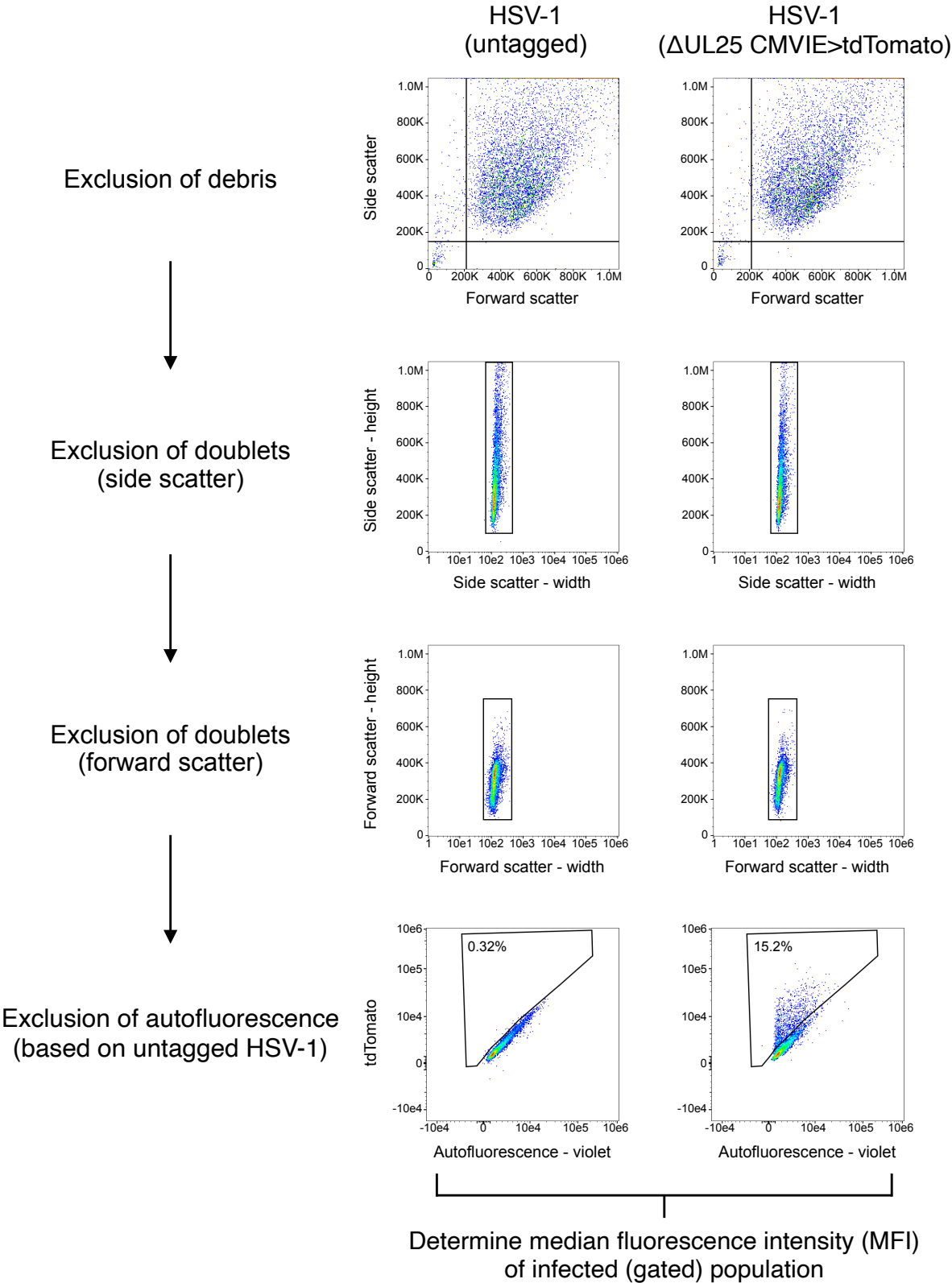

Supplement: Supplemental Figures — Figures S1 to S6. [file mbio.01445-24-s0004.pdf]
