# Supplementary material for: Leveraging biotin-based proximity labeling to identify cellular factors governing early alphaherpesvirus infection
Source: mBio. 2024 Jul 2;15(8):e01445-24. doi: 10.1128/mbio.01445-24 (PMC11323796; doi:10.1128/mbio.01445-24)
Supplement: Legends — for Figures S1-S6. [file mbio.01445-24-s0005.pdf]

SUPPLEMENTAL FIGURE LEGENDS

Supplemental Figure 1. Immunoblot of BioID2-fusion proteins from lysates of PK15 cells infected with BioID2-tagged or untagged (-) PRV at MOI:3 for 6 or 18 hrs.

Supplemental Figure 2. Log_{10} spectral counts for viral proteins detected by PRV-BioID2 proximity labeling in HeLa cells at 18 hpi. Zero spectral counts are shown as a blank cell.

Supplemental Figure 3. Reduced expression of WASH components increases permissivity to infection. (**A**) Immunoblot of RPE lysates that were transduced for strumpellin KO with guide RNAs (gRNAs) 1 – 4 individually or pooled gRNAs 1-4, or a non-targeting scrambled gRNA control (NT). The strumpellin exon (out of 29 total) targeted by each gRNA is indicated. (**B**) Immunoblot of RPE lysates that were transduced for FAM21C KO with gRNAs. The FAM21C exon (out of 31 total) targeted by each gRNA is indicated. (**C**) Fraction of RPE cells emitting nuclear fluorescence (% infected) analyzed by flow cytometry following infection with HSV-1 single-round reporter at 6 hpi. (**D**) Fraction of RPE cells emitting fluorescence (% infected) analyzed by flow cytometry following infection with wild-replication-competent HSV-1 at 6 hpi. Error bars are standard deviations. ** $p < 0.01$, *** $p < 0.001$, **** $p < 0.0001$ (unpaired t-tests).

Supplemental Figure 4. Kinetics of reporter gene expression from the HSV-1 single-round reporter in WT and zyxin-KO RPE cells at 4-8 hpi determined by flow cytometry. Mean values are plotted with standard deviation (n=3). Two replica experiments are shown. * $p < 0.05$, ** $p < 0.01$, *** $p < 0.001$ (unpaired t-tests).

Supplemental Figure 5. Depletion of zyxin increases PRV and HSV-1 infection. (**A**) Fraction of wild-type (WT), zyxin-KO (KO), and rescue (Res) cells emitting fluorescence (% infected) analyzed by flow cytometry following infection with replication competent PRV at MOI:30 for 6 hpi. (**B**) Fraction of cells emitting fluorescence following

infection with replication competent HSV-1 at MOI:5 for 6 hpi. * $p < 0.05$, ** $p < 0.01$ (paired t-tests). Each data point is the mean of internal triplicates, with the overall mean across 3 replicas indicated with standard error.

Supplemental Figure 6. Flow cytometric gating strategy used to determine percent infection and median fluorescence intensity (MFI) using untagged HSV-1 and the HSV-1 single-round reporter. Representative flow plots for infection of WT RPE cells are shown. Cells were gated to exclude debris and doublets. The violet channel was used to detect and exclude autofluorescent cells. Only cells that were above the threshold for tdTomato, as determined by the untagged infection control, were counted as infected.
